# Dietary Diversity Is Positively Associated with Deviation from Expected Height in Rural Nepal^1^,^2^,^3^

**DOI:** 10.3945/jn.115.220137

**Published:** 2016-06-15

**Authors:** Laura K Busert, Melissa Neuman, Eva A Rehfuess, Sophiya Dulal, Jayne Harthan, Shiva Shankar Chaube, Bishnu Bhandari, Harry Costello, Anthony Costello, Dharma S Manandhar, Naomi M Saville

**Affiliations:** 4 Institute for Medical Informatics, Biometry, and Epidemiology, Ludwig-Maximilians-Universität München, Munich, Germany;; 5 Institute for Global Health, University College London, London, United Kingdom; and; 6 Mother and Infant Research Activities, Kathmandu, Nepal

**Keywords:** dietary diversity, child growth, child growth recovery, child growth faltering, stunting, conditional growth, Nepal

## Abstract

**Background:** Recent research has highlighted the need for additional studies on the nutrition input required to stabilize growth.

**Objective:** Our objective was to examine the association between dietary diversity and conditional growth in children aged 0–89 mo.

**Methods:** We analyzed cohort data from 529 mothers and children living in a remote and food-insecure region in the mountains of Nepal. Children were aged 0–59 mo at baseline and were followed up after 9 and 29 mo. Conditional growth was calculated as the deviation from the expected height-for-age difference (HAD) trajectory based on previous measures of HAD and the pattern of growth in the population. Dietary diversity was assessed with the use of a count of the foods consumed from 7 food groups in the previous 7 d. The association between dietary diversity and conditional growth during the 2 follow-up periods (of 9 and 20 mo, respectively) was estimated with the use of ordinary least-squares regressions.

**Results:** Prevalence of stunting and absolute height deficits was very high and increased over the course of the study. At the last measurement (age range 29–89 mo), 76.5% were stunted and the mean ± SD HAD was −11.7 ± 4.6 cm. Dietary diversity was associated positively with conditional growth in the later (May 2012–December 2013) but not the earlier (July 2011–May 2012) growth period. Children’s ages ranged from 0 to 59 mo in July 2011, 9 to 69 mo in May 2012, and 29 to 89 mo in December 2013. After adjustment, increasing the dietary diversity by one food group was associated with a 0.09 cm (95% CI: 0.00, 0.17 cm) increase in conditional growth in the second growth period.

**Conclusions:** Increasing dietary diversity for children reduces the risk of stunting and improves growth after growth faltering. Future efforts should be directed at enabling families in food-insecure areas to feed their children a more diverse diet.

## Introduction

Undernutrition was the underlying cause of 3.1 million or 45% of all deaths in children <5 y of age in 2011 (1). One key indicator of chronic undernutrition is low length or height for age or stunting, which indicates failure to reach linear growth potential (2, 3). In 2014, 23.8% of children <5 y of age worldwide had a height-for-age *z* score (HAZ)^7^ of >2 SDs below the WHO reference for their age and sex and thus were considered to be stunted (4). The largest number of children affected by stunting, 69 million, live in south-central Asia (1). In Nepal, 41% of children <5 y of age are stunted, with the highest prevalence found in mountainous areas in the western region (59.5%) (5).

The short- and long-term consequences of childhood stunting are well documented. In childhood, low height for age is a risk factor for increased morbidity and mortality from infectious diseases (6, 7). Poor growth in the first 2 y of life is associated with reduced cognitive ability and school performance in adolescence and shorter height and reduced income in adulthood; intergenerational effects, in particular, reduced offspring birth weight, are also well documented (8).

Growth faltering is caused by an insufficient intake of macro- and micronutrients in combination with frequent infections early in a child’s life (9). It is generally accepted that recovery from growth retardation after 2 y of age is only possible if the child has few infections and consumes a diet that is adequate in nutrient requirements (10, 11). Recent research has highlighted how difficult it is for children to catch up after a period of insufficient growth: although earlier analyses had identified improvements in HAZs in some children in low-resource settings, this more recent analysis found no improvement in average height deficit in children <5 y of age (12). This lack of agreement within the literature highlights the need for additional evidence that uses robust measures of child growth and nutritional intake to identify the factors needed to reduce stunting prevalence in low-resource settings.

A critical aspect of a diet’s adequacy is diversity. In poor populations in low-income countries, diets are often unbalanced and composed primarily of starchy staples, whereas consumption of animal products is low, and the availability of fruits and vegetables is dependent on the season (13). Consumption of a higher number of food groups has been shown to improve the likelihood of meeting the required amounts of macronutrients and most micronutrients in children (14–17). Extensive research has demonstrated the association of dietary diversity with attained HAZ at different ages in childhood (13, 15, 17–19), but we are not aware of any longitudinal study examining the association of dietary diversity with conditional growth, which is the change in height over time not predicted by previous heights. Because dietary diversity is a critical determinant of child nutritional status, our objective was to examine the association of dietary diversity and conditional growth over 2 growth periods in children <8 y of age. Such evidence can help inform policies and programs to improve the health and prospects of young children who have experienced growth faltering. We hypothesized that dietary diversity was positively associated with conditional growth. For the present study, we used data from a cohort study in Jumla district in the mountains of the midwestern region of Nepal. Jumla is one of the remotest districts, with a Human Development Index ranked 70th of 75 districts in 2001 (20). Adults in this district have only 1.6 y of schooling on average, and adult literacy rates are very low (20). Food insecurity is a serious concern for much of the population, and the prevalence of stunting in children <5 y was one of the highest in the country at 74% in 2004 (20). Although these data are >10 y old, it is likely that this prevalence is still among the highest in Nepal.

## Methods

### 

#### Study setting and participant selection.

Our study was conducted in the 3 neighboring geopolitical clusters (village development committees) of Dillichaur, Chhumchaur, and Patarasi, which were identified by the district nutrition rehabilitation center as having a particularly high prevalence of severe acute malnutrition. Within these 3 village development committees, all households with ≥1 children aged ≤59 mo at baseline in July 2011 were eligible for enrollment. We enrolled all households in which the family either could be found at home or presented for anthropometric measurements at a central location within a village. If there was >1 child <5 y of age in the household, we enrolled the youngest child. Follow-up examinations were conducted in May 2012, when the children were aged 9–69 mo, and December 2013, when the children were aged 29–89 mo. Of the 689 households enrolled at baseline, 655 were followed up in May 2012, with 626 attending a second follow-up (9% loss to follow-up). Of those children who could not be followed up, 6 had died between baseline and the first follow-up, and 2 had died between the first and second follow-ups.

#### Data.

At each data collection point, anthropometric measurements of the child and mother were taken and a questionnaire in the Nepali language was administered to the mother or the primary caregiver. The questionnaire included proximal determinant variables of child undernutrition, such as feeding practices and any recent occurrence of infectious diseases, as well as socioeconomic determinants at the maternal and household level. Several variables used in this analysis were not collected at each time point, but only at the first measurement, or the first and last measurements. A summary of the variables used in this analysis and the time of their measurement are provided in **Supplemental Table 1**. In order to ensure good mutual understanding between interviewers and interviewees, data collectors were recruited locally and questions were asked in the local Jumli dialect. Before each survey, the data collectors underwent rigorous training on questionnaire administration and anthropometric measurement; collectors were supervised during data collection by experienced field staff. Paper questionnaires were used for the baseline questionnaire and the first follow-up. In the second follow-up, we collected data on mobile devices with the use of digital questionnaires programmed with Open Data Kit open source application (21).

The interviewers received oral informed consent from the mothers of the children before beginning the interviews and taking anthropometric measurements. The study protocol received ethical approval from the Nepal Health Research Council.

#### Child nutritional status and growth.

Supine length (for all children during the first data collection, and for children <24 mo of age and <87 cm in height at the first follow-up) and standing height (for children ≥24 mo of age or ≥87 cm in height) were measured to the nearest completed millimeter with the use of height and length boards accurate to 1 mm. Children’s weight was measured with the use of baby scales accurate to 2 decimal places (for children weighing <20 kg) or adult scales accurate to 1 decimal place (for children weighing ≥20 kg). Growth deficits were assessed with the use of the absolute height-for-age difference (HAD) (12), which is the difference between attained height and the median age- and sex-specific height obtained from the growth reference. We used the WHO 2006 standard for children <5 y of age (22) and the WHO 2007 reference for school-aged children (23) to compute HAD (in centimeters) and HAZ (in SD units).

Our primary outcome of interest was conditional growth, which represents the change in HAD between measurements conditional on the starting values of HAD. We chose this method of analysis because it allowed us to control for all time-invariant factors that affect growth. We calculated conditional growth for 2 growth periods, i.e., from baseline (July 2011; age range: 0–59 mo) to the first follow-up 9 mo after baseline (May 2012; age range: 9–69 mo), and from the first to the second follow-up 20 mo after the first follow-up (December 2013; age range: 29–89 mo), with the use of ordinary least-squares (OLS) regressions, with the HAD at the end of the growth period as the outcome and prior measures of HAD and age at the beginning of the growth period as predictors. To obtain conditional growth 1 (from baseline to the first follow-up) we calculated OLS as follows:


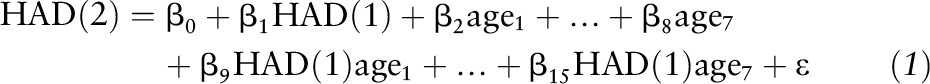


HAD (1) and HAD (2) denote HAD at baseline (July 2011) and the first follow-up (May 2012), respectively. We included indicators for 7 age categories at baseline (relative to a reference category of 0–5 mo) to allow for nonlinearity in the association of age with HAD. We also included an interaction between age and HAD at baseline to adjust for different patterns of growth within age groups. Conditional growth 1 is the studentized residual from this regression and thus represents the variance-standardized difference between the observed HAD at the first follow-up and the HAD predicted by the model. An analogous procedure was used to compute conditional growth 2 (from the first to the second follow-up). Conditional growth captures both downward and upward deviations from the growth trajectory predicted by the child’s age, the previous HAD, and the pattern of growth in that population (24).

#### Dietary diversity.

Dietary diversity was assessed with the use of a dietary diversity score, a count of food groups consumed over a given reference period (25). At each data collection, we recorded the mother’s recall of the foods from a list of 15 groups the child had consumed in the previous 7 d. We followed WHO guidance in grouping these foods into the following 7 food groups (26): *1*) starchy staples (grains and root tubers), *2*) legumes, *3*) dairy products, *4*) flesh foods (meat and fish), *5*) eggs, *6*) vitamin A–rich fruits and vegetables (leafy green vegetables, yellow fruits, and yellow vegetables), and *7*) other fruits and vegetables. A simple dietary diversity score was calculated by counting the number of food groups the child had consumed in the past 7 d. At baseline, 22.7% of the children in our sample were still breastfed exclusively, whereas at the first follow-up, all children had started eating complementary foods. We therefore decided that for the purpose of this study the best indicator of the child’s dietary diversity in the respective growth period was the dietary diversity score at the end of that period, i.e., at the first follow-up for conditional growth 1, and at the second follow-up for conditional growth 2.

#### Covariates.

Potential confounding factors in the association between dietary diversity and conditional growth were identified with the use of a directed acyclic graph (DAG) (**Supplemental Figure 1**) and the software DAGitty (27). DAGs serve to represent visually assumed causal relations between exposures, outcomes, and covariates and are used to identify potential sources of bias in observational health research (28). We determined a minimally sufficient adjustment set that contained the following factors: household wealth, household food insecurity, crowding, maternal height, maternal education, child care during illness, continued breastfeeding, child infections, general care, child age, and sex. Wherever possible, we identified actual variables or proxies from our data set. Because we did not have information for several covariates at the first follow-up, we decided to use the most proximal information for the respective growth period, i.e., covariates at baseline for growth period 1 and covariates at the second follow-up for growth period 2.

A wealth index was constructed with the use of principal component analysis from a number of variables, including toilet ownership, selected household assets (such as radio or electricity), land ownership, and income from a family member who had migrated for work (particularly for wild-mushroom harvesting). Households were categorized into wealth-quintiles, from poorest (quintile 1) to least poor (quintile 5). Household food insecurity was assessed with the use of the Household Food Insecurity Access Prevalence, derived from answers to the Household Food Insecurity Access Scale questionnaire (29). The Household Food Insecurity Access Prevalence categorizes households into food secure and mildly, moderately, and severely food insecure according to the severity and frequency with which they have experienced anxiety about food supply, been forced to consume low-quality foods, and had an insufficient quantity of food over the previous 30 d. Crowding was assessed based on the number of household members per sleeping room. The mother’s height was measured at baseline with the use of height boards and remeasured at the second follow-up if she was <22 y of age and could have still grown since the first measurement. We adjusted for maternal education in terms of years of schooling completed. Child infection was based on the occurrence of diarrhea in the previous 14 d and acute respiratory infection (ARI) on the previous day, as reported by the mother. If the child had suffered from diarrhea, we asked the mother questions while following UNICEF guidance (30) about the quantity of food and liquids she had fed during and after the illness and whether she had given the child any oral rehydration salts. Children who had been fed less than usual or who had not been given any oral rehydration salts were classified as not having received appropriate care. We further adjusted for whether the child was still breastfed, as well as for the child’s age and sex.

#### Statistical analysis.

For our analytical sample we used all observations with complete data. In order to ensure good data quality, we excluded cases in which any of the following criteria applied: absolute HAD or absolute HAZ was >5 SDs of the HAD or HAZ, respectively, for the population, absolute changes in HAZ were >3 SDs between baseline and the first follow-up (9 mo) or >4 SDs for the period from the first to the second follow-up (20 mo), or longitudinal length or height measures were implausible (e.g., length or height at follow-up less than length at baseline). Of the 689 observations, we retained 529 with complete data in the analytical sample for conditional growth 1 (77% of the initial sample) and 515 for conditional growth 2 (75%). In the sample for conditional growth 1, excluded children had taller mothers who were more likely to care appropriately for their children in case of diarrhea and had a higher dietary diversity score than did the children retained in the study. For conditional growth 2, we found no differences between the analytical sample and excluded observations.

In the descriptive analysis, we characterized the dietary composition at each data collection. We estimated the crude and mutually adjusted associations between conditional growth and covariates with the use of OLS regressions. We further conducted 3 sensitivity analyses. First, because the appropriate use of indicators to characterize longitudinal growth patterns in children has been the subject of debate (31–33), we repeated our analysis with the use of HAZ instead of HAD. Second, because the association of dietary diversity with changes in growth in stunted children was the focus of our research, we repeated our analysis in a subsample of children with HAZ <−2 at the beginning of the respective growth period (i.e., at baseline for conditional growth 1 or at the first follow-up for conditional growth 2). Our third sensitivity analysis was performed because we had observed that there were differences between the analytical sample and excluded observations. To test whether our findings might have been biased through missing data, we repeated our analysis after including observations with missing values as additional categories.

We used an α level of 0.05 for all statistical tests. All analyses were performed with the use of Stata IC 12.1.

## Results

At baseline, when children were 0–59 mo old, almost two-thirds of the children had an HAZ of <−2; prevalence of stunting rose to 76.5% at the last measurement, when children were 29–89 mo old. Over the course of the study, mean HAD dropped from −7.2 ± 4.9 cm at baseline to −11.7 ± 4.6 cm at the second follow-up, and mean HAZ dropped from −2.5 ± 1.4 to −2.8 ± 1.1 (**Table 1**). Compared with both baseline and the second follow-up, mean weight-for-age and weight-for-length or -height *z* scores were significantly lower (at −2.1 ± 1.0 and −1.0 ± 1.2, respectively) at the first follow-up. Levels of infection measured by incidence of diarrhea and ARI were higher in July 2011 than in December 2013. Of those children who had diarrhea in the 14 d preceding the interview, only a minority received appropriate care. As expected, the percentage of households reporting any food insecurity was higher at baseline when data were collected in the premonsoon months than at the second follow-up when data were collected in December after the harvest. In contrast, the mean dietary diversity score was slightly lower in the winter season in December than during the summer months of July and May, probably because of the dearth of fresh fruits, vegetables, and dairy foods available in the winter season. Only a small percentage of the mothers (7.6%) had ever been to school.

**TABLE 1:** Characteristics of the study population by time of data collection^1^

	Baseline (July 2011)	First follow-up (May 2012)	Second follow-up (December 2013)
Participants, *n*	529	529	515
Dietary diversity score^2^	3.6 ± 1.5	3.9 ± 1.1	3.4 ± 1.1
Nutritional status			
HAD, cm	−7.2 ± 4.9	−9.1 ± 4.8	−11.7 ± 4.6
HAZ	−2.5 ± 1.4	−2.7 ± 1.3	−2.8 ± 1.1
WHZ	−0.5 ± 1.1	−1.0 ± 1.2	−0.2 ± 1.0
BMIZ^3^	—	−1.5 ± 2.2	−0.4 ± 0.8
WAZ^4^	−1.8 ± 1.1	−2.1 ± 1.0	−1.9 ± 0.9
Sex			
M	53.9	—	—
F	46.1	—	—
Age, mo			
0–5	19.1	—	—
6–8	9.3	—	—
9–11	7.2	5.9	—
12–17	18.9	19.8	—
18–23	8.7	19.1	—
24–35	17.4	23.4	21.2
36–47	12.9	15.7	34.8
48–59	6.6	11.2	19.2
60–71	—	4.9	15.0
72–83	—	—	8.9
84–91	—	—	1.0
Diarrhea and quality of care			
No diarrhea	65.2	—	88.3
Diarrhea and good care	4.0	—	2.9
Diarrhea and inappropriate care	30.8	—	8.7
ARI^5^	2.6	—	1.4
Child is breastfed	85.3	—	27.0
Child is exclusively breastfed	22.7	0.0	0.0
Wealth index^6^	0.0 ± 1.3	—	—
Household food insecurity			
Food secure	41.4	—	68.9
Mild food insecurity	24.2	—	7.0
Moderate food insecurity	18.3	—	13.8
Severe food insecurity	16.1	—	10.3
Maternal height, cm	149.4 ± 5.3	—	—
Household members per sleeping room	—	—	3.1 ± 1.5
Mother’s years of schooling	—	—	0.5 ± 1.8

1 Values are means ± SDs or percentages. ARI, acute respiratory infection; BMIZ, BMI-for-age *z* score; HAD, height-for-age difference; HAZ, height-for-age *z* score; WAZ, weight-for-age *z* score; WHZ, weight-for-height *z* score.

2 The number of food groups consumed in the 7 d preceding the interview out of 7 food groups; at baseline, this excludes 120 children who had not started complementary foods.

3 For children aged >60 mo, BMIZs were calculated instead of WHZs. Missing observations—baseline: 5; first follow-up: 1; and second follow-up: 5.

4 Missing observations—baseline: 6; first follow-up: 6; and second follow-up: 1.

5 On the day preceding the interview as reported by the mother.

6 Constructed with the use of principal component analysis from a number of variables, including toilet ownership, selected household assets (such as radio and electricity), land ownership, and income from a family member who had migrated for work (particularly for wild-mushroom harvesting).

**FIGURE 1:**
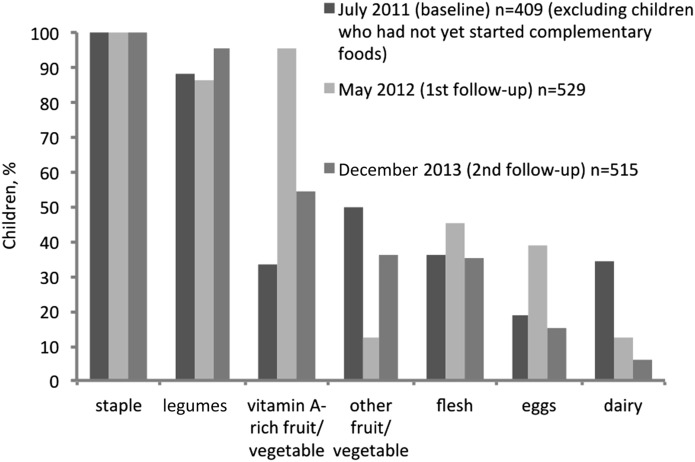
Percentage of children who had consumed each food group ≥1 time in the 7 d preceding the survey, by time of data collection. Age ranges were the following—baseline: 0–59 mo; first follow-up: 9–69 mo; and second follow-up: 29–89 mo.

Children’s dietary composition varied substantially across data collection periods (**Figure 1**). In May 2012, 95.0% had consumed vitamin A–rich fruits or vegetables, but at the other 2 measurements, the overall consumption of fruits and vegetables was considerably lower (54.4% and 33.3%, respectively). Intake of animal-source foods was relatively low across all time periods and lowest in December 2013. Staple foods and legumes were a constant component of the diet in almost all children who had started eating complementary foods.

The crude and mutually adjusted associations between conditional growth and covariates are listed in **Table 2**. Crude associations of dietary diversity with conditional growth were positive in both growth periods, but significant only in the second period (20-mo duration; age range: 9–69 mo at the first follow-up and 29–89 mo at the second follow-up). Maternal height was associated positively with conditional growth in both growth periods. In the first growth period (9-mo duration; age range: 0–59 mo at baseline and 9–69 mo at the first follow-up), children with ARI on the day preceding the interview had lower conditional growth than children who were not sick. A higher number of household members per sleeping room was associated with low conditional growth in the second growth period. (The results of the regressions used to obtain conditional growth estimates from HAD and HAZ are presented in **Supplemental Tables 2** and** 3**).

**TABLE 2:** Crude and mutually adjusted associations between conditional growth (HAD) and covariates in Nepalese children over the 2 growth periods^1^

	Conditional growth 1 (*n* = 529)				Conditional growth 2 (*n* = 515)			
	Crude associations		Adjusted associations		Crude associations		Adjusted associations	
	B (95% CI)	*P*	B (95% CI)	*P*	B (95% CI)	*P*	B (95% CI)	*P*
Dietary Diversity Score^2^	0.06 (−0.02, 0.14)	0.13	0.04 (−0.04, 0.12)	0.29	0.09 (0.02, 0.17)	0.02	0.09 (0.00, 0.17)	0.04
Maternal height, cm	0.03 (0.01, 0.05)	<0.01	0.03 (0.01, 0.05)	<0.01	0.02 (0.01, 0.04)	<0.01	0.03 (0.01, 0.04)	<0.01
Household members per sleeping room	−0.05 (−0.11, 0.00)	0.06	−0.04 (−0.10, 0.02)	0.18	−0.07 (−0.13, 0.01)	0.01	−0.08 (−0.14, −0.02)	0.01
Mother’s years of schooling	0.01 (−0.04, 0.06)	0.79	−0.01 (−0.06, 0.04)	0.63	−0.03 (−0.08, 0.02)	0.22	−0.05 (−0.10, 0.00)	0.06
Female	0.13 (−0.04, 0.30)	0.14	0.12 (−0.06, 0.29)	0.19	−0.07 (−0.24, 0.11)	0.46	−0.10 (−0.27, 0.08)	0.29
Age,^3^ mo								
0–5	Ref		Ref		—		—	
6–8	−0.01 (−0.35, 0.34)	0.97	−0.07 (−0.42, 0.28)	0.70	—		—	
9–11	−0.01 (−0.39, 0.37)	0.95	0.02 (−0.36, 0.41)	0.92	Ref		Ref	
12–17	0.00 (−0.28, 0.28)	1.00	0.00 (−0.28, 0.28)	1.00	−0.00 (−0.41, 0.41)	1.00	0.07 (−0.34, 0.48)	0.74
18–23	0.01 (−0.34, 0.37)	0.95	0.01 (−0.35, 0.36)	0.97	−0.00 (−0.41, 0.41)	1.00	0.11 (−0.31, 0.52)	0.61
24–35	0.00 (−0.28, 0.29)	0.98	0.02 (−0.27, 0.31)	0.89	−0.00 (−0.41, 0.40)	1.00	0.08 (−0.33, 0.48)	0.71
36–47	0.00 (−0.31, 0.31)	0.99	0.04 (−0.27, 0.36)	0.80	−0.00 (−0.43, 0.43)	1.00	0.08 (−0.35, 0.51)	0.72
48–59	0.00 (−0.39, 0.39)	0.99	0.03 (−0.36, 0.42)	0.87	0.00 (−0.45, 0.45)	1.00	0.09 (−0.37, 0.54)	0.70
60–71	—		—		−0.00 (−0.55, 0.55)	0.99	0.13 (−0.43, 0.69)	0.65
Diarrhea and quality of care	
No diarrhea	Ref		Ref		Ref		Ref	
Diarrhea and good care	0.07 (−0.37, 0.52)	0.75	0.08 (−0.37, 0.53)	0.73	0.30 (−0.21, 0.82)	0.25	0.31 (−0.22, 0.83)	0.25
Diarrhea and inappropriate care	0.07 (−0.12, 0.26)	0.47	0.09 (−0.11, 0.28)	0.37	0.00 (−0.31, 0.31)	1.00	0.03 (−0.28, 0.35)	0.84
ARI^4^	−0.60 (−1.13, −0.06)	0.03	−0.59 (−1.14, −0.04)	0.04	−0.13 (−0.89, 0.62)	0.73	−0.16 (−0.93, 0.61)	0.69
Child is breastfed	—		—		0.07 (−0.12, 0.27)	0.46	0.10 (−0.11, 0.31)	0.33
Wealth quintile	
1	Ref		Ref		Ref		Ref	
2	−0.01 (−0.27, 0.27)	0.94	−0.01 (−0.29, 0.27)	0.93	−0.06 (−0.33, 0.21)	0.67	−0.07 (−0.35, 0.20)	0.59
3	0.10 (−0.16, 0.36)	0.45	0.10 (−0.19, 0.39)	0.51	−0.06 (−0.33, 0.20)	0.64	−0.12 (−0.39, 0.15)	0.37
4	0.21 (−0.07, 0.48)	0.14	0.21 (−0.09, 0.51)	0.17	0.10 (−0.18, 0.38)	0.46	0.08 (−0.21, 0.36)	0.60
5	0.24 (−0.02, 0.51)	0.08	0.22 (−0.09, 0.52)	0.17	0.08 (−0.20, 0.35)	0.58	0.03 (−0.26, 0.32)	0.83
Household food insecurity	
Food secure	Ref		Ref		Ref		Ref	
Mild food insecurity	−0.10 (−0.32, 0.12)	0.37	−0.03 (−0.25, 0.20)	0.81	−0.07 (−0.42, 0.28)	0.69	0.03 (−0.33, 0.38)	0.88
Moderate food insecurity	−0.11 (−0.35, 0.13)	0.37	0.00 (−0.26, 0.26)	0.98	−0.08 (−0.34, 0.18)	0.55	0.01 (−0.25, 0.28)	0.93
Severe food insecurity	−0.16 (−0.41, 0.10)	0.23	0.06 (−0.24, 0.35)	0.70	−0.06 (−0.36, 0.23)	0.66	0.13 (−0.18, 0.44)	0.41

1 First growth period (conditional growth 1; 9-mo duration) is from baseline (age range: 0–59 mo) to first follow-up (age range: 9–69 mo). Second growth period (conditional growth 2; 20-mo duration) is from first follow-up (age range: 9–69 mo) to second follow-up (age range: 29–89 mo). Conditional growth is studentized residual representing deviation from expected linear growth in the prior interval based on HAD; conditional growth 1 is from baseline (July 2011) to first follow-up (May 2012), and conditional growth 2 is from first follow-up to second follow-up (December 2013). Adjusted *R*^2^ for both conditional growth 1 and conditional growth 2 was 0.01. ARI, acute respiratory infection; HAD, height-for-age difference; Ref, reference.

2 The number of food groups consumed in the 7 d preceding the interview out of 7 food groups.

3 At the beginning of the growth period, i.e., age at baseline for conditional growth 1 and age at first follow-up for conditional growth 2.

4 On the day preceding the interview as reported by the mother. For conditional growth 1, the information was collected at baseline (beginning of the growth period); for conditional growth 2, it was collected at the second follow-up (end of the growth period).

After adjustment, the association between dietary diversity and conditional growth was unchanged, indicating that an increase in dietary diversity by 1 food group/wk was associated with a 0.09 cm (95% CI: 0.00, 0.17 cm) higher conditional growth. Only ARI and maternal height were associated with conditional growth in the first growth period. Besides dietary diversity, the mother’s height and the number of household members per sleeping room were associated with conditional growth in the second growth period.

The results of our first sensitivity analysis are presented in **Supplemental Table 4**. Using HAZ to calculate conditional growth would not have changed our major findings. **Supplemental Table 5** shows the results of our second sensitivity analysis. We found that in a subsample of initially stunted children, the association between dietary diversity and conditional growth was not significant. In the third sensitivity analysis, we found that including observations with missing categorical covariates did not change the results of our primary analysis.

## Discussion

In a cohort study in a poor and remote area of Nepal with a very high prevalence of stunting, we examined the association between dietary diversity and conditional growth in children over 2 growth periods. We found evidence that dietary diversity was associated positively with conditional growth in the second period, but not in the first growth period studied. In addition, we found conditional growth to be associated positively with maternal height in both growth periods, and associated negatively with ARI and the number of household members per sleeping room in the first and second growth periods, respectively. Two sensitivity analyses showed that our results were robust to missing data and across different measurements of growth.

It was not possible to compare directly effects with findings from previous studies because of differences in measures of both dietary diversity and child growth. However, the positive association that we found in 1 of the 2 growth periods is in line with findings from cross-sectional studies investigating the association between some measure of dietary diversity and attained HAZ (13, 15, 17–19). Therefore, the lack of an association in the first growth period is surprising. One explanation could be that our assumption that a dietary diversity score based on a 7-d recall was a good proxy for nutrient intake in the preceding growth period is not accurate. At baseline, 85.3% of the children were still breastfed and 22.7% had not yet started eating complementary foods. We can therefore assume that other factors, such as the frequency of breastfeeding and the mother’s nutritional status during lactation (9), largely determined the child’s growth in that period. This argument is supported by a study that found the association of dietary diversity and attained HAZ to be stronger in nonbreastfed children in Nepal (13). Unfortunately, no information on the breastfeeding status at the first follow-up was available.

We also assessed the association between dietary diversity and conditional growth within the subsample of children who were stunted at the beginning of the respective growth period (*n* = 382 for conditional growth 2, compared with *n* = 515 in the full sample of children). Within this subsample, the association with dietary diversity was positive but not significant (*P* = 0.14). We believe that our nonsignificant finding was a result of the small sample limiting our power to detect this association as statistically significant.

The negative trend between the mother’s years of schooling and conditional growth is surprising, because it contradicts previous studies that found maternal education to reduce the risk of offspring stunting (13, 34). Very few children in our population had mothers with any formal education (<8%), and these children had a different growth trajectory than their peers: at baseline they had a considerably higher mean HAZ (−1.9 compared with −2.5), which dropped with the next measurement but was still higher than that of the other children (−2.2 compared with −2.7); at the second follow-up they reached the same mean HAZ as the rest of the study population (−2.8). Unfortunately, we cannot provide a potential mechanism explaining this trend, and the possibility that this association is only due to statistical chance cannot be ruled out. Nevertheless, it may be worth assessing the role of the mother’s educational attainment in child nutrition in future research in similar populations.

A number of limitations may have influenced our results. First, we did not have data available to control for all potential sources of confounding and bias as identified in the DAG. One factor that we could not account for in our model was general care. General care comprises many different aspects of child care, e.g., responsive feeding, child stimulation, and activity (9), that might have reduced the effect of dietary diversity on conditional growth toward the null. On the same note, we cannot rule out the fact that residual confounding by socioeconomic status or household sanitary environment may have biased our results. Second, our study was limited by some loss to follow-up and missing data. However, attrition was low, and a sensitivity analysis revealed that including observations with missing categorical covariates would not have changed our results. One limitation of this method is that we could only test the potential impact of missing values for those observations in which the outcome and all continuous covariates were available (*n* = 549 for growth period 1 and *n* = 524 for growth period 2) and not for those lost to follow-up. A third limitation is that our analyses of the 2 growth periods are not entirely comparable. First, the duration of the 2 growth periods (9 mo and 20 mo) is different. Second, because several variables such as ARI and diarrhea were not collected at the first follow-up, we had to use the information at baseline (the beginning of the growth period) for the analysis of growth period 1 and at that at the second follow-up (the end of the growth period) for growth period 2. The analyses assume that one-off measures, whether taken at the beginning or end of the period, form a useful proxy for the entire period under study, which could be inaccurate but is not unusual in epidemiological studies. However, bearing in mind the difficulty in obtaining data from remote Himalayan communities of this kind, we feel this makes the best use of the available data.

Despite these limitations, our study contributes to understanding the association between dietary diversity and child growth. Our study is novel because we collected longitudinal data and focused on deviations from the expected growth trajectory rather than attained height or HAZ. An important strength of our study is our analytical approach: by analyzing residuals, we could control for all time-invariant factors that affect child growth, such as the structural or environmental factors (e.g., access to health care, cleanliness of drinking water, or location of the village) or factors at the family and household level (e.g., the family’s socioeconomic status, parental education, or the child’s position within the family) that either were not included in the analysis or were insufficiently captured in the covariates used. Analyzing 2 subsequent growth periods also allowed us to compare the effects of dietary diversity on child growth at different ages in the child’s life. A further strength of this study is that we used data from a population that is heavily burdened by stunting and at the same time situated in a remote location that is very difficult to reach. Because of the terrain and the simple housing, it was difficult to even find flat surfaces suitable for anthropometric measurements. Heavy rainfall during the monsoon, deep snow in winter, and mothers engaged in agricultural activities away from their homes further complicated measurements and made finding all eligible mothers and their children difficult. Although these circumstances could have led to less accurate measurements or fewer individuals sampled and followed-up, every effort was made to ensure the best data quality possible: anthropometry was repeated until measurements diverged by <1% (weight) or <2% (length or height), and mothers were visited in the fields if their chores otherwise would not have allowed them to participate in the study. Especially in such areas in which the burden is so high, it is important that efforts to assess and address undernutrition reach such remote populations, not just those that are convenient to study.

In conclusion, our study provides further evidence that eating a more diverse diet is beneficial for child growth. Future efforts should be directed at examining how families in very poor and food-insecure areas such as this can be empowered to feed their children a more diverse diet throughout the year. The inconsistent associations of dietary diversity with child conditional growth between the growth periods indicate that at a younger age, factors other than dietary diversity are crucial in determining how children deviate from their growth trajectory. These findings point to the need for promoting age-appropriate feeding practices in order to prevent stunting and to increase children’s chances of recovery from growth faltering.

## Footnotes

7 Abbreviations used: ARI, acute respiratory infection; DAG, directed acyclic graph; HAD, height-for-age difference; HAZ, height-for-age *z* score; OLS, ordinary least-squares.
